# Dissipation behavior and risk assessment of four pesticides in shiitake mushrooms from cultivation to processing

**DOI:** 10.1038/s41538-026-00975-5

**Published:** 2026-07-06

**Authors:** Jingyun Liang, Shuai Guan, Mengmeng Yan, Junhua Liu, Liping Fang, Jiangsheng Mao, Zhan Dong, Kai Cui, Changhan Zhang

**Affiliations:** 1 Institute of Agricultural Products Quality Safety and Standards, Shandong Academy of Agricultural Sciences, Jinan, China; 2 College of Food Science and Bioengineering, Tianjin Agricultural University, Tianjin, China

**Keywords:** Biochemistry, Chemistry, Environmental sciences

## Abstract

Through field trials, this study comprehensively investigated the dissipation, metabolism, processing factors (PFs), and risk assessment of dinotefuran, imidacloprid, pyraclostrobin, thiamethoxam, and their metabolites in shiitake mushrooms from cultivation to postharvest processing. In the laboratory, common postharvest industrial and household processing methods were simulated, including washing, boiling, pickling, stir-frying, sauce processing, hot-air processing, and crisp processing. Understanding the fate of residues is crucial for ensuring food safety and safeguarding human health. The limit of quantification (LOQ) for 6-chloronicotinic acid, thiamethoxam and clothianidin was 10 μg/kg, while the LOQs for other analytes were 2 μg/kg. Field trials showed that the degradation of the four pesticides followed first-order kinetics, with half-lives ranging from 1.1 to 1.8 days. After washing, boiling, pickling, stir-frying, and sauce processing, the concentrations of all pesticides—except for a metabolite of pyraclostrobin (BF-500-3)—were reduced to varying degrees (PF < 1). Hot air processing led to 4.3- and 4.6-fold increases in the concentrations of dinotefuran and 6-chloronicotinic acid, respectively, while the concentrations of all other pesticides decreased (PF < 1). Crisp processing increased the concentration of all pesticide residues by a factor of 2.0 to 4.0. Additionally, the content of BF-500-3 increased significantly by 2.9–4.9 times after washing, boiling, pickling, and stir-frying. However, it decreased after hot air processing and sauce processing. The risk quotient values of all pesticides were below 1, indicating that both chronic and acute dietary risks were acceptable. This study provides valuable data for the control of pesticide residues in processed products, which has both theoretical and practical significance in ensuring the quality and safety of shiitake mushrooms and protecting human health.

**Figure:**
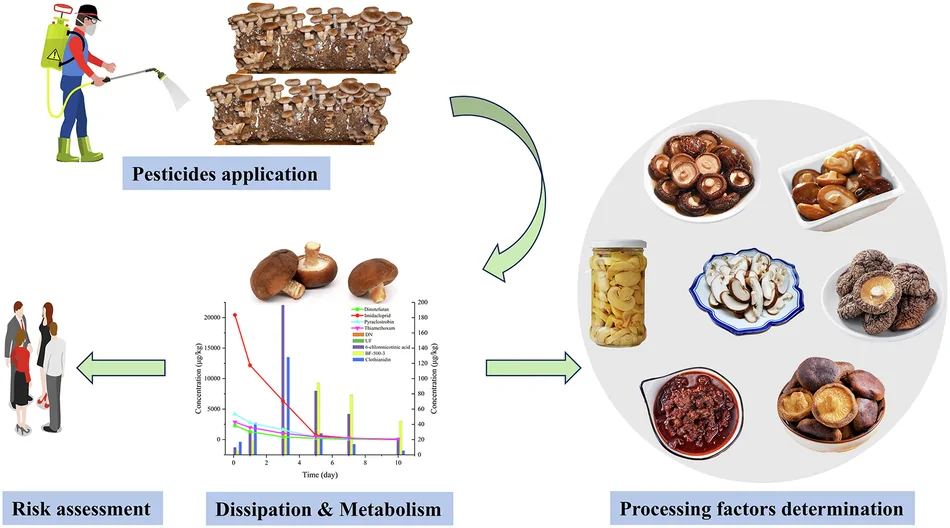

## Introduction

Shiitake (*Lentinus edodes*) mushroom is one of the most widely cultivated and versatile edible and medicinal fungi in China. It is primarily grown through artificial cultivation and ranks as the second most consumed edible mushroom worldwide^1,2^. Shiitake mushrooms play a significant role in various domains, including food, medicine, and ecological sustainability^3,4^. These mushrooms are rich in polysaccharides, proteins, vitamins, dietary fiber, and essential minerals, and have demonstrated biological activities including immune enhancement, tumor prevention and treatment, antiviral effects, and antioxidant properties^3–6^. With the advancement and widespread adoption of cultivation techniques such as sawdust substrate farming, shiitake mushroom production in China has increased rapidly. In 2022, output reached 12.955 million tons, accounting for 98.3% of global production^7^. China has thus become the world’s largest producer and exporter of shiitake mushrooms^3,8^.

Under high-temperature and high-humidity cultivation conditions, shiitake mushrooms are highly susceptible to various diseases and pests. To manage these threats, multiple insecticides and fungicides are commonly applied during production. However, the use of such pesticides inevitably leads to residue accumulation, posing potential risks to human health and environmental safety, as well as causing substantial economic losses to the industry^9,10^. Pesticide residues in edible fungi have been identified as a significant concern by several researchers. For instance, a study conducted in the Czech Republic detected 21 different pesticide residues and their metabolites in 49 samples of edible mushrooms^11^. Residues of more than one pesticide were found in 45% of the samples. The most frequently quantified pesticide residues included prochloraz and its three metabolites(58% of the samples above LOQ), metrafenone (32.2% of the samples with quantified residues), carbendazim and benzalkonium chlorides (22.6% of the samples with quantified residues). Exceedance of the legal limits was found for permethrin(dried Jew’s ears) and thiabendazole (fresh enoki mushrooms). Similarly, Li et al. investigated pesticide residue levels in edible fungi from central and western China, finding that only 52 out of 354 samples were free of detectable residues^9^. Carbendazim had the highest detection rate (70.9%), followed by acephate (13.0%) and procymidone (7.3%), and the detection concentrations were 0.0002–2.7316, 0.0248–0.4985, 0.1807–0.3928 mg/kg, respectively. Research on the degradation, migration, and dietary risk assessment of pesticides in crops is therefore critical for controlling residue levels and safeguarding consumer health.

In recent years, research on the dissipation behavior, processing factors (PFs), and risk assessment of pesticides in shiitake mushrooms has remained notably scarce; furthermore, the range of pesticides evaluated is narrow, and the vast majority of existing studies focus solely on pre-harvest dissipation kinetics. Liu et al. examined the degradation dynamics of carbendazim, thiabendazole, procymidone, λ-cyhalothrin, β-cyfluthrin, and bifenthrin in shiitake mushrooms^1^. Sun et al. analyzed the dissipation patterns of fipronil and avermectin in shiitake mushrooms^12^. Du et al. studied the dissipation behavior of beta-cypermethrin, pyriproxyfen, avermectin, and diflubenzuron under different application methods^13^. Chrysayi-Tokousbalides et al. conducted residue evaluations of famoxadone and trifloxystrobin in cultivated mushrooms^14^. Dinotefuran, imidacloprid, pyraclostrobin, and thiamethoxam are pesticides registered for use in China^15^. They are also the most extensively utilized pesticides during the shiitake mushroom cultivation process. In China, there are 37 registered plant protection products that are allowed to be used on edible fungi and contain these four active ingredients, which account for approximately half of all registered plant protection products^15^. However, to date, limited research has focused on the degradation and migration of these four pesticides and their metabolites in shiitake mushrooms. Notably, metabolites of these four pesticides display distinct toxicological profiles, with several demonstrating higher toxicity than their parent compounds—thereby amplifying human health risks associated with dietary intake of contaminated edible plants^16^. The metabolic pathways of dinotefuran in plants encompass desmethylation, nitro-reduction, and hydrolysis^17^. DN and UF are the primary metabolites of dinotefuran. These two metabolites exhibit toxicity levels comparable to dinotefuran and demonstrate greater environmental mobility and persistence^18^. The metabolism of imidacloprid in rats was rapid. Extensively metabolized through oxidative cleavage, hydroxylation and conjugation. The main urinary metabolites were 6-chloronicotinic acid^17^. In several regulatory jurisdictions, the residue definition adopted for maximum residue limit (MRL) setting comprises imidacloprid and its metabolite 6-chloronicotinic acid^19–21^. BF-500-3, the desmethoxy metabolite of pyraclostrobin, is considered to exhibit toxicity comparable to that of the parent compound. In the United States and Canada, the MRL for pyraclostrobin in crops includes both pyraclostrobin and its metabolite BF-500-3^22^. The major reaction involved in the biotransformation of thiamethoxam is the cleavage of the oxadiazine ring to the corresponding nitroguanidine compound. In the oral administration test in rats, 22 metabolites were isolated from the excreta. The quantitatively most important metabolite was clothianidin, which accounted for about 10% of the dose^17^. Clothianidin and thiamethoxam are both neonicotinoid insecticides with similar toxicological profiles; moreover, clothianidin is a metabolic transformation product of thiamethoxam^23^. In addition, upon checking, in China, the MRL values of imidacloprid and thiamethoxam in fresh Agaricus bisporus are 2.0 and 0.5 mg/kg, respectively. In Japan, the MRL values of dinotefuran, thiamethoxam, and clothianidin in shiitake mushrooms are 0.5, 0.7, and 0.05 mg/kg, respectively. In the European Union, the MRL values of imidacloprid, pyraclostrobin, thiamethoxam, and clothianidin in cultivated fungi are 0.01, 0.02, 0.01, and 0.01 mg/kg, respectively^15,21,24^. Therefore, when evaluating the degradation behavior and residue risks of such pesticides in agricultural commodities, it is essential to consider the combined contributions of both the parent compounds and their relevant metabolites^25^.

Fresh shiitake mushrooms have a high moisture content and are highly susceptible to spoilage and oxidative discoloration when stored under ambient conditions. Postharvest, they are commonly subjected to various processing methods such as washing, drying, freezing, stir-frying, pickling, and boiling prior to consumption. Among these, drying, freezing, and pickling are the most effective for extending shelf life^1^. These processes transform fresh shiitake mushrooms into a range of commercial products, including dried shiitake, pickled shiitake, and cooked shiitake, with shiitake sauce and crisps being particularly popular. Numerous studies indicate that food processing can influence pesticide residues in agricultural commodities^10,26–28^. However, different processing techniques variably affect both nutritional quality and sensory attributes, as well as alter pesticide residue profiles. For instance, among various drying methods, freeze-drying and hot air drying are reported to best preserve nutritional components^29–31^. Washing has been shown to significantly reduce pesticide residues^32^, whereas stir-frying may lead to residue concentration or thermal transformation products. Drying can increase residue concentrations due to water loss, potentially posing health risks to consumers^3,33^. Moreover, certain pesticides may degrade into more toxic metabolites during processing^28,34,35^. The extent of pesticide reduction depends on the physicochemical properties of the pesticides and the specific processing conditions, such as temperature, duration, and method (freezing, drying, heating)^27^. Therefore, it is essential to conduct a comprehensive investigation into the fate of these four pesticides and their metabolites throughout the cultivation and processing of shiitake mushrooms, as well as their associated dietary risks, to safeguard human health. To date, PF values for these pesticides in shiitake mushrooms have not been reported in the literature.

This study conducted a field experiment to investigate the fate of four plant protection products—dinotefuran, imidacloprid, pyraclostrobin, and thiamethoxam—and their metabolites in shiitake mushrooms throughout the entire process from cultivation to postharvest processing. The objectives were threefold: (1) to examine the dissipation dynamics of these pesticides in shiitake mushrooms under field conditions and determine their dissipation half-lives; (2) to evaluate the residue behavior of four different types of pesticides and their metabolites in fresh shiitake mushrooms under diverse processing conditions (temperature, duration, and heat treatment methods)—including washing, boiling, pickling, stir-frying, sauce processing, hot air processing, and crisp processing—and to estimate corresponding processing factors; and (3) to assess dietary health risks for both children and the general population based on the experimental findings. The results provide initial insights into the residue profiles of the four pesticides and their metabolites across the farm-to-consumption chain. Moreover, this study serves as a practical reference for growers to optimize pesticide application, informs consumers of evidence-based strategies for reducing pesticide residues on shiitake mushrooms, and establishes a scientific foundation for shiitake mushroom product manufacturers to implement residue control measures throughout post-harvest processing.

## Results

### Method validation

The determination of pesticide residues, including dinotefuran, imidacloprid, pyraclostrobin, thiamethoxam, and their respective metabolites, was conducted using the QuEChERS method^36^. Validation results, summarized in Table S1, demonstrate that the analytical method achieved excellent performance. All target compounds exhibited strong linearity across the tested concentration range, with correlation coefficients (R²) ranging from 0.9928 to 0.9999, meeting the criteria specified in the SANTE/11312/2021 v2 guideline. The LOQs of 6-chloronicotinic acid, thiamethoxam and clothianidin were 10 μg/kg, whereas the LOQs for other analytes were 2 μg/kg. Matrix effects ranged from −73.1% to +85.4%, indicating either suppression or enhancement of the instrumental response due to components in the shiitake mushroom matrix. To mitigate such effects, matrix-matched calibration curves were employed for quantification. Method accuracy and precision were assessed through recovery experiments in three representative matrices: fresh shiitake mushrooms, dried shiitake mushrooms, and shiitake mushroom sauce. At three spiking levels, recoveries for all compounds ranged from 74% to 106%, with relative standard deviations (RSDs) from 2.0% to 15.0%. These outcomes comply with the acceptance criteria, confirming the reliability and robustness of the validated method in accordance with current regulatory guidelines^37^.

### Residue dissipation kinetics

**Fig. 1:**
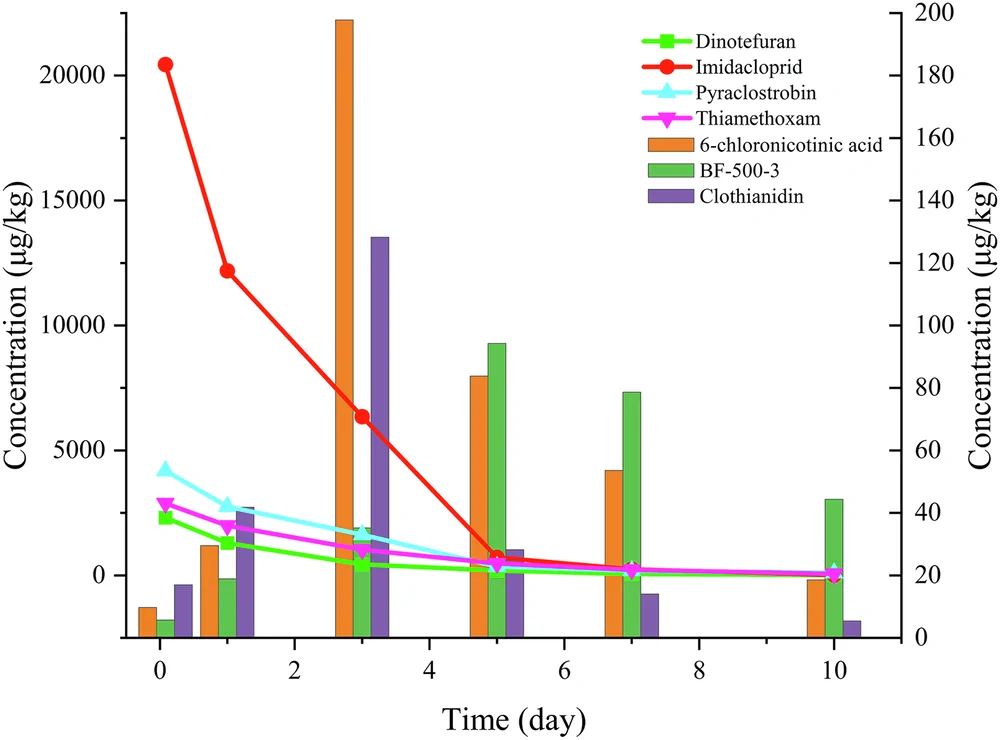
Dissipation kinetics of four pesticides and their metabolites in shiitake mushrooms.

Studying the dissipation behavior of pesticides under field conditions is a crucial approach for evaluating the environmental risks associated with pesticide use and for guiding the safe and rational application of pesticides in agricultural production^32,38^. Figure 1 illustrates the dissipation patterns of four pesticides—dinotefuran, imidacloprid, pyraclostrobin, and thiamethoxam—and five of their metabolites in shiitake mushrooms under controlled greenhouse conditions. Initial residue levels were recorded at 2310.57 μg/kg for dinotefuran, 20444.23 μg/kg for imidacloprid, 4183.28 μg/kg for pyraclostrobin, and 2884.83 μg/kg for thiamethoxam. The dissipation dynamics of these four pesticides followed first-order kinetics, with correlation coefficients ranging from 0.9659 to 0.9987, yielding half-lives of 1.4, 1.1, 1.7, and 1.8 days, respectively (Table 1). Three days after application, degradation rates exceeded 60% for all four pesticides, increasing to approximately 90% by day 5. Notably, dinotefuran exhibited the fastest degradation, exceed 80% degradation within three days, resulting in the lowest residual concentration in shiitake mushrooms. Previous studies have reported half-lives of dinotefuran on wolfberry, tomato, and paddy rice as 1.4, 2.5, and 3.0 days, respectively^39–41^;imidaclopridhalf-lives on citrus and beans, were 1.9 and 2.1 days, respectively^42,43^; pyraclostrobin half-lives on cucumber, greenhouse dill, cowpea, and watermelon were 8.2, 1.62, 1.5, and 3.2 days, respectively^44–47^; and thiamethoxam half-lives on peach, spinach, and greenhouse chrysanthemum were 4.9, 1.6, and 8.2 days, respectively^24,48,49^. This research was conducted under controlled greenhouse conditions, with a constant temperature of 20 °C and humidity of 80%. In contrast, field trials in the wild are subject to the influence of light, temperature, rainfall and other climatic factors, making it impossible to ensure uniform experimental conditions. As a result, the half-lives of pesticides on different crops vary. The experimental results show that the dissipation rates of these four pesticides in shiitake mushrooms under greenhouse conditions are faster than those in other crops under field conditions in the wild. This indicates that various factors—including crop species, environmental temperature, humidity, light intensity, application rate, and pesticide formulation—can influence the dissipation behavior of pesticides in crops, leading to variations in dissipation half-lives across different crop types^32,45,47^.

**Table 1:** The residue concentrations, dissipation rates, dissipation dynamic equations, correlation coefficient (*R*^2^) and half-lives (*t*_1/2_) of four pesticides in shiitake mushrooms

Time (days)	Dinotefuran		Imidacloprid		Pyraclostrobin		Thiamethoxam	
	Residue (μg/kg)	Dissipation rates (%)	Residue (μg/kg)	Dissipation rates (%)	Residue (μg/kg)	Dissipation rates (%)	Residue (μg/kg)	Dissipation rates (%)
2 h	2310.57	/	12187.21	/	4183.28	/	2884.83	/
1 d	1299.07	43.8	6351.11	47.9	2757.45	34.1	1989.03	31.0
3 d	443.61	80.8	3842.85	68.5	1632.12	61.0	1027.38	64.4
5 d	197.10	91.5	721.93	94.1	354.39	91.5	477.96	83.4
7 d	53.48	97.7	245.61	98.0	189.13	95.5	222.72	92.3
10 d	14.12	99.4	14.41	99.9	95.23	97.7	61.34	97.9
Dynamic equations	*C*_t_ = 2250.5e^-0.513t^		*C*_t_ = = 16791e^-0.659t^		*C*_t_ = 4071.5e^-0.404t^		*C*_t_ = 3081.4e^-0.384t^	
*R*^2^	0.9959		0.9699		0.9863		0.9987	
*t*_1/2_ (day)	1.4		1.1		1.7		1.8	

The residues of DN, UF, 6-chloronicotinic acid, BF-500-3, and clothianidin are presented in Fig. 1. Of the five metabolites analyzed, neither DN nor UF was detected in any of the field trial samples. This could be attributed to the relatively low initial concentration of dinotefuran in shiitake mushrooms (2.31 mg/kg). The initial concentration of 6-chloronicotinic acid was also extremely low. Subsequently, it increased gradually and reached its peak concentration of 197.81 μg/kg on day 3. Clothianidin reached its peak concentration of 128.24 μg/kg on day 3. However, BF-500-3 reached a peak level of 94.28 μg/kg on day 5. By day 5, about 2.5% of pyraclostrobin was transformed into BF-500-3, and approximately 1.4% of imidacloprid and 2.6% of thiamethoxam were metabolized into 6-chloronicotinic acid and clothianidin by day 3. Li et al. investigated the dissipation patterns of dinotefuran, UF, and DN in wolfberry^39^, which is consistent with the findings of this study, as both metabolites remained at low levels throughout the experimental period. Similarly, the degradation trends of 6-chloronicotinic acid, BF-500-3 and clothianidin are consistent with previous findings in citrus, greenhouse-grown dill and spinach^42,45,48^.

The parent pesticides exhibit different dissipation behaviors, as evidenced by differences in dissipation rates and residual concentrations. Some pesticides dissipate rapidly, while others dissipate slowly. Some have high residual concentrations, while others have low residual concentrations. Nevertheless, their dissipation trends are basically consistent: as time passes, the residual concentrations of all parent pesticides gradually decline until they are completely dissipated. The metabolite content changes gradually as the parent pesticides dissipate. Initially, the parent pesticides have not decomposed, and no metabolites have been produced. As time elapses, the metabolites are generated as the parent pesticides are metabolized and decomposed, and their content gradually rises. On the one hand, because of the gradual degradation of the parent pesticide, and on the other hand, since the metabolite itself also dissipates, the content of the metabolite will not increase indefinitely. Therefore, the content of the metabolites gradually accumulates to a peak over time. After reaching the peak, the concentration gradually decreases until the metabolites are completely dissipated. Overall, the dissipation trends of all metabolites are consistent, but their dissipation rates and residual concentrations vary.

### Residue changes and PFs during processing

**Fig. 2:**
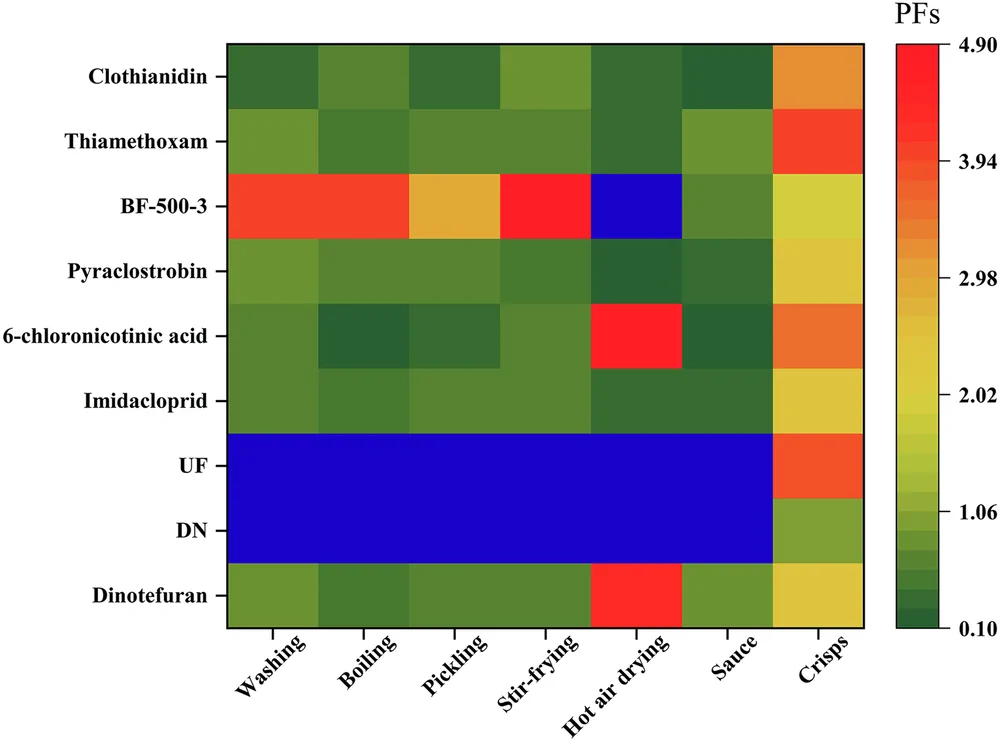
Heatmap of the processing factors (PFs) of pesticides in shiitake mushrooms.

The concentration of pesticides during food processing is influenced by multiple factors, including initial pesticide concentrations, crop harvest time, processing techniques, food types, and pesticide properties. As shown in Table 1, the concentration of pesticide residues in crops varies significantly at different harvest times. If the harvest time is too late, pesticide residues in crops are relatively low, and residues in processed products cannot be effectively detected. If the harvest time is too early, the mushrooms' growth period is short, and the caps have not fully developed, making it difficult to collect a sufficient number of samples for research. Therefore, to ensure that the samples used for processing experiments contain quantifiable and representative pesticide residues, this study collected fresh shiitake mushrooms 3 days after pesticide application. The water content in food is a crucial indicator of its type, as it determines the food's relative weight after drying. Foods with a high water content will experience a reduction in weight after drying, which is also a significant factor influencing PFs. After testing, the water content of fresh shiitake mushrooms is found to be 78.89%. Moreover,research has indicated that the behavior of pesticide residues is affected by their physicochemical characteristics—such as volatility, water-octanol partition coefficient (log Kow), solubility, melting point, and degradation point—which in turn influence the efficacy of processing methods^50^. Table S2 summarizes the physicochemical properties of dinotefuran, imidacloprid, pyraclostrobin, thiamethoxam, and clothianidin. In this study, household and industrial processing methods were simulated under laboratory conditions to evaluate the changes in residues of dinotefuran, imidacloprid,pyraclostrobin, thiamethoxam, and their metabolites across various processing techniques, including washing, boiling, pickling, stir-frying, hot-air drying, sauce processing, and crisp processing. Residue levels are presented in Table 2 and Fig. 2. UF and DN were only detected in the products after crisp processing and were not detected in other processed products, owing to their parent pesticide's relatively low initial concentrations. Therefore, the concentration changes in these two metabolites during processing will not be discussed further. Except for BF-500-3, washing reduced pesticide residues in shiitake mushrooms by 13.2% to 61.0%, with PFs ranging from 0.4 to 0.9. For boiling, the pesticide residue removal rate ranged from 27.9% to 79.6%, with PFs between 0.2 and 0.7. In pickling, the removal rate varied from 25.3% to 61.0%, with PFs ranging from 0.4 to 0.7. For stir-frying, the removal rates of other pesticides were observed between 21.6% and 47.7%, with corresponding PFs from 0.5 to 0.8. During hot air drying, BF-500-3 was not detected, and the PFs of all other compounds ranged from 0.1 to 4.6. Notably, the concentrations of dinotefuran and 6-chloronicotinic acid increased by 4.3- and 4.6-fold, compared to pre-drying levels, whereas the concentrations of other compounds in dried shiitake mushrooms decreased significantly, with removal rates of 61.5% to 72.3%. In sauce processing, the concentrations of all pesticides decreased significantly, resulting in PFs ranging from 0.2 to 0.9 and removal rates of 9.0% to 77.3%. After crisp processing, the concentrations of all pesticides increased markedly, with PFs ranging from 2.0 to 4.0. The results of the statistical test further indicated that, with the exception of dinotefuran during the sauce processing, the PF values of all pesticides exhibited significant differences under different treatment conditions(Table 2, *P* < 0.05).

**Table 2:** Residue levels and processing factors (PFs) of four pesticides and five metabolites during shiitake mushrooms processing

Processing	Residues (μg/kg)		Dinotefuran			Residues (μg/kg)	DN			Residues (μg/kg)	UF		
			PFs	*P*	Removal rate %		PFs	*P*	Removal rate %		PFs	*P*	Removal rate %
Raw	443.61 ± 11.6		-	-	-	n.d.	-	-	-	n.d.	-	-	-
Washing	369.51 ± 4.1		0.8	0.009	16.7	n.d.	-	-	-	n.d.	-	-	-
Boiling	231.14 ± 1.2		0.5	0.001	47.9	n.d.	-	-	-	n.d.	-	-	-
Pickling	331.27 ± 5.7		0.7	0.004	25.3	n.d.	-	-	-	n.d.	-	-	-
Stir-frying	293.48 ± 4.6		0.7	0.002	33.8	n.d.	-	-	-	n.d.	-	-	-
Hot air drying	1918.11 ± 1.5		4.3	<0.001	−332.4	n.d.	-	-	-	n.d.	-	-	-
Sauce	403.85 ± 21.9		0.9	0.102	9.0	n.d.	-	-	-	n.d.	-	-	-
Crisps	1124.23 ± 26.0		2.5	0.001	−153.4	3.05	-	-	-	7.60	-	-	-
**Processing**			**Imidacloprid**				**6-chloronicotinic acid**				**Pyraclostrobin**		
	**Residues (μg/kg)**	**PFs**		*P*	**Removal rate %**	**Residues (μg/kg)**	**PFs**	*P*	**Removal rate %**	**Residues (μg/kg)**	**PFs**	*P*	**Removal rate %**
Raw	3842.85 ± 107.9		-	-	-	197.81 ± 7.4	-	-	-	1632.12 ± 31.5	-	-	-
Washing	2301.54 ± 48.3		0.6	0.002	40.1	143.36 ± 2.2	0.7	0.003	27.5	1417.29 ± 82.9	0.9	0.028	13.2
Boiling	1837.87 ± 30.8		0.5	0.001	52.2	40.29 ± 2.1	0.2	<0.001	79.6	1188.10 ± 55.9	0.7	0.01	27.2
Pickling	2380.32 ± 70.0		0.6	0.001	38.1	77.76 ± 2.4	0.4	0.002	60.7	1138.74 ± 17.3	0.7	0.002	30.2
Stir-frying	2413.65 ± 19.5		0.6	0.001	37.2	129.13 ± 7.4	0.7	0.002	34.7	854.05 ± 23.2	0.5	0.002	47.7
Hot air drying	1215.96 ± 77.1		0.3	0.001	68.4	900.24 ± 7.1	4.6	<0.001	−355.1	120.57 ± 5.9	0.1	<0.001	92.6
Sauce	1812.54 ± 45.8		0.5	<0.001	52.8	45.53 ± 3.8	0.2	<0.001	77.0	593.61 ± 11.0	0.4	0.001	63.6
Crisps	9735.47 ± 89.9		2.5	<0.001	−153.3	696.08 ± 8.1	3.5	<0.001	−251.9	3842.47 ± 117.2	2.4	0.001	−135.4
**Processing**			**BF-500-3**				**Thiamethoxam**				**Clothianidin**		
	**Residues (μg/kg)**		**PFs**	*P*	**Removal rate %**	**Residues (μg/kg)**	**PFs**	*P*	**Removal rate %**	**Residues (μg/kg)**	**PFs**	*P*	**Removal rate %**
Raw	35.20 ± 1.2		-	-	-	1027.38 ± 60.5	-	-	-	128.24 ± 6.5	-	-	-
Washing	146.01 ± 5.3		4.1	0.001	−314.8	773.98 ± 20.0	0.8	0.013	24.7	55.59 ± 3.7	0.4	0.006	61.0
Boiling	143.63 ± 4.9		4.1	<0.001	−308.0	535.88 ± 14.3	0.5	0.008	47.8	75.61 ± 4.7	0.6	0.003	41.0
Pickling	101.55 ± 6.5		2.9	0.002	−188.5	657.33 ± 40.3	0.6	0.023	36.0	50.04 ± 1.3	0.4	0.003	61.0
Stir-frying	172.36 ± 2.6		4.9	<0.001	−389.6	726.32 ± 22.6	0.7	0.024	29.3	100.50 ± 4.2	0.8	0.046	21.6
Hot air drying	n.d.		-	-	-	395.08 ± 16.1	0.4	0.005	61.5	35.52 ± 0.9	0.3	0.002	72.3
Sauce	25.32 ± 1.1		0.7	0.002	28.1	783.96 ± 19.8	0.8	0.033	23.7	29.15 ± 0.9	0.2	0.001	77.3
Crisps	69.62 ± 3.6		2.0	0.002	−97.8	4075.16 ± 59.1	4.0	0.001	−296.7	409.04 ± 7.4	3.2	0.001	−219.0

*P*-values from PF at different processing using paired *t*-test at 95% probability.

The experimental results indicate that, with the exception of BF-500-3, the concentrations of all pesticides and their metabolites decreased to varying extents after washing, boiling, pickling, stir-frying, and sauce processing. However, after crisp processing, the concentrations of all compounds increased compared to their initial levels. The removal efficiencies of the target pesticides varied significantly, attributable to differences in their physicochemical properties. Specifically, the log Kow value is a key determinant of a compound’s polarity and partitioning behavior. Pesticides with higher log Kow values exhibit greater lipophilicity and hydrophobicity^51^, whereas those with lower log Kow values and higher water solubility are more readily removed through aqueous-based processes such as washing, boiling, and pickling^29^, a trend consistent with the findings of this study. Dinotefuran, imidacloprid, thiamethoxam, and clothianidin all have low log Kow values (<1), while pyraclostrobin has the highest (log Kow = 3.99). Consequently, pyraclostrobin showed the lowest removal rate after washing. Notably, despite its high log Kow value, pyraclostrobin exhibits a low melting point, which renders it more prone to degradation under high-temperature conditions like boiling, stir-frying, and sauce processing. In contrast, during crisp processing, the PF for all target pesticides in freeze-dried mushrooms exceeded unity (PF > 1), indicating concentration enhancement. Specifically, pesticide concentrations increased two- to fourfold relative to their pre-processing levels, resulting in significantly elevated residue levels compared with those observed in fresh mushrooms. Notably, DN and UF were only detected in the products after crisp processing, while they were not detected in any other processed products. Freeze-drying is a low-temperature, high-vacuum dehydration process that eliminates over 95% of the moisture from shiitake mushroom samples. Significantly, this technique maintains the chemical integrity of pesticide residues as it neither causes thermal nor oxidative degradation nor promotes their leaching or matrix-bound transformation. As a result, the significant reduction in water content causes a proportional rise in residue concentration on a mass basis. This enrichment effect, which is due to moisture removal and results in increased pesticide concentrations in the dried matrix, has been consistently reported in previous studies^52–54^. Similarly, DN and UF could be detected in freeze-dried shiitake mushrooms, yet they were not detectable in other products. This was also attributed to the increased concentration of these substances in the matrix caused by water evaporation. Thus, the potential enrichment of pesticide residues must be rigorously addressed in the safety assessment of freeze-dried shiitake mushrooms. After hot-air drying, the concentration of dinotefuran and 6-chloronicotinic acid was higher than before drying. This phenomenon might be attributed to the high solubility of dinotefuran in water (39,800 mg/L). Prior to the drying process, dinotefuran would dissolve in the moisture present in the mushroom substrate. As moisture gradually evaporated during drying, dinotefuran became increasingly concentrated. In addition, due to the high initial concentration of imidacloprid and its low solubility, it may further dissipate during the drying process. This leads to a significantly higher concentration of 6-chloronicotinic acid than that before drying. In contrast, the concentrations of imidacloprid, pyraclostrobin, thiamethoxam, and clothianidin in the dried samples were lower than those in the raw samples, likely because these pesticides are susceptible to volatilization and thermal degradation under elevated temperatures, leading to reduced residue levels^54,55^. This suggests that drying temperature and duration can differentially influence pesticide residue concentrations. Additionally, test results revealed a notable trend: the concentration of the metabolite BF-500-3 decreased only after hot-air drying and sauce processing, but increased significantly after other processing methods. This may be attributed to the breakdown of pyraclostrobin into BF-500-3 under certain conditions. The varying behaviors of these metabolites, combined with the lack of Log Kow data, indicate that the underlying mechanisms require further investigation.

### Dietary risk assessment of pesticides in shiitake mushrooms

In this study, the PF was below 1 for most processing procedures. Furthermore, the Food and Agriculture Organization (FAO)/World Health Organization (WHO) report on edible fungus consumption data exclusively covers dried products, not fresh ones^56^. In particular, there is a shortage of LP data on fresh mushrooms, which makes it impossible to calculate the acute risk quotient. Consequently, common household processing methods such as washing, boiling, pickling, stir-frying, and sauce-based processing were excluded from the exposure assessment. Only dietary exposure risk assessments for dried shiitake mushrooms and shiitake crisps—processed via hot air drying and crisp processing techniques—were performed. Based on pesticide residue levels in dried and crispy shiitake mushrooms across different sampling intervals and the consumption patterns of various population groups, the chronic and acute dietary exposure risks were assessed. The results of the dietary risk assessment for different pesticides are presented in Tables S3 and S4. In the chronic dietary risk assessment of dried shiitake mushrooms, the calculated RQc values were <0.001–0.096 for children and <0.001–0.034 for the general population, both substantially below the threshold of 1. For acute dietary risk, the RQa values were <0.001–0.007 in children and <0.001–0.009 in the general population, also well below 1. Notably, imidacloprid posed the highest chronic dietary risk associated with dried mushroom consumption, with RQc values of 0.096 for children and 0.034 for the general population. The next highest risk was posed by dinotefuran. In shiitake crisps, the chronic dietary risk assessment yielded RQc values ranging from <0.001 to 0.803 for children and from <0.001 to 0.0.287for the general population. The corresponding acute risk values (RQa) ranged from <0.001 to 0.135 for children and from <0.001 to 0.175 for the general population, remaining under the safety threshold in all cases. Due to the high initial concentration of imidacloprid, when consuming shiitake crisps, the imidacloprid in the samples 2 h after application contributed the greatest chronic risk, with RQc values of 0.803 for children and 0.287 for the general population. The RQc values are close to the safety threshold of 1. When consuming shiitake crisps, pyraclostrobin exhibits the highest acute risk, with the RQa values of 0.135 for children and 0.175 for the general population. Overall, both the chronic and acute dietary risks associated with pesticide residues in shiitake mushrooms remain within acceptable limits.

Previous studies have indicated that dietary exposure to pesticides from consuming shiitake mushrooms generally does not pose significant health risks^1^. However, based on the findings of this study, when consuming dried shiitake mushrooms, the chronic dietary risks associated with imidacloprid and dinotefuran are higher than those of other pesticides. This is because the initial imidacloprid content is relatively high, and the PFs of dinotefuran during the drying process of shiitake mushrooms are also relatively high, resulting in increased pesticide residues in the final dried products. Similarly, for shiitake mushroom crisps, imidacloprid poses a higher chronic dietary risk than other pesticides, attributable to both elevated initial residue levels and its relatively low ADI. Therefore, potential health concerns associated with consuming dried shiitake mushrooms and shiitake mushroom crisps should not be overlooked. Furthermore, certain pesticides may undergo metabolic transformation in the human body, generating toxicological metabolites that could act synergistically with parent compounds, thereby enhancing overall toxicity^57^. Moreover, during the processing of dried shiitake mushrooms and shiitake mushroom crisps, pesticide residue levels may concentrate, potentially increasing dietary exposure risks. Therefore, greater attention should be directed toward monitoring and regulating pesticide residues in shiitake mushrooms and their processed products to safeguard public health and ensure food safety.

## Discussion

To the best of our knowledge, this study represents the first comprehensive evaluation of the dissipation, metabolism, PFs, and dietary risks associated with dinotefuran, imidacloprid, pyraclostrobin, thiamethoxam, and their metabolites throughout the entire process from cultivation to postharvest handling in shiitake mushrooms. The results demonstrate that the dissipation kinetics of all target pesticides followed a first-order kinetic model, with half-lives ranging between 1.1 and 1.8 days. The dissipation trends of all metabolites are consistent. Over time, the content of these metabolites gradually accumulates to a peak. Subsequently, the concentration gradually decreases until the metabolites are completely dissipated.

For the target pesticides, various processing methods, including washing, boiling, pickling, stir-frying, and sauce processing, can remove pesticide residues from shiitake mushrooms to varying degrees, as indicated by PFs <1. In contrast, crisps processing significantly concentrates the pesticide residues and all metabolites. The levels of pesticide residues are 2.0 to 4.0 times higher than in fresh shiitake mushrooms, suggesting a greater risk to the human diet. Hot air drying reduces most pesticide residues; however, dinotefuran and 6-chloronicotinic acid show the opposite effect, with their concentrations 4.3 and 4.6 times higher than before hot air drying. It is essential to strengthen the monitoring of shiitake mushroom crisps and dried shiitake mushroom products and optimize the processing parameters to minimize the residue concentration. Additionally, BF-500-3's performance is quite distinctive. After almost all processing methods, its concentration shows a concentration phenomenon, with PFs > 1. When pyraclostrobin is applied in the cultivation of shiitake mushrooms, special attention should also be paid to the issue of metabolite contamination that it may cause to the processed products. The pesticide residues in shiitake mushrooms during processing are significantly influenced by the physicochemical properties of individual pesticides. Specifically, pesticides with lower log Kow and higher water solubility tend to be more readily removed during household processing. However, in the production of shiitake mushroom crisps, freeze-drying technology leads to water sublimation, resulting in a marked enrichment and concentration of residual pesticides in the dried matrix.

Chronic and acute risk assessments indicate that neither children nor the general population is exposed to significant dietary risks from consuming dried shiitake mushrooms or shiitake mushroom crisps, as the RQc and RQa values are below 1. This study elucidates the dissipation behavior of multiple pesticides and their metabolites under field conditions and tracks residue changes during various processing methods, thereby providing scientific references for the rational use of pesticides and the protection of human health.

## Methods

### Chemicals and reagents

**Fig. 3:**
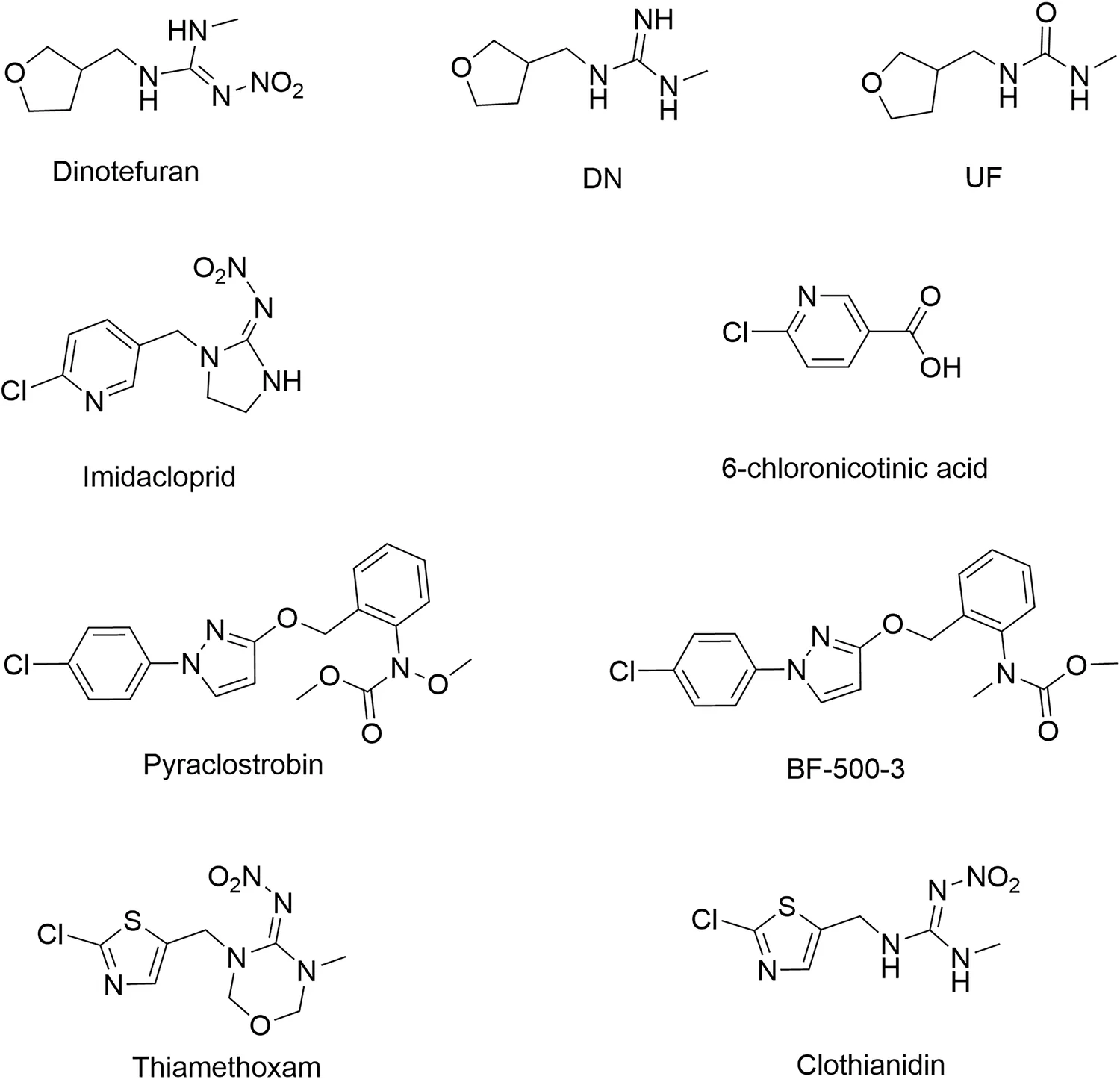
Chemical structures of dinotefuran (and metabolites DN, UF), imidacloprid (and metabolite 6-chloronicotinic acid), pyraclostrobin (and metabolite BF-500-3), and thiamethoxam (and metabolite clothianidin).

Individual standard substances of dinotefuran, imidacloprid, pyraclostrobin, and thiamethoxam, along with their metabolites (DN, UF, 6-chloronicotinic acid, BF-500-3, and clothianidin) (purity ≥95.0%), were obtained from Shandong Daizong Chemical Technology Co., Ltd. (Jinan, China) (Fig. 3). Chromatographic-grade acetonitrile and formic acid were purchased from Thermo Fisher Scientific (Shanghai, China), while ammonium acetate was sourced from Sigma-Aldrich (MO, USA). Buffer-salt-mixture containing 4 g anhydrous magnesium sulfate (MgSO_4_), 1 g sodium chloride (NaCl), 1 g trisodium citrate dihydrate, 0.5 g disodium hydrogencitrate sesquihydrate was acquired from Agilent Technologies (CA, USA). Centrifuge tubes containing 150 mg of primary secondary amine (PSA) and 900 mg of MgSO_4_ were provided by Agilent Technologies (CA, USA). Nylon syringe filters (0.22 μm) were supplied by Bonna-Agela Technologies Co., Ltd. (Tianjin, China). Stock solutions of all target pesticides were prepared in acetonitrile at a concentration of 1000 mg/L and stored at –20 °C until use. Working standard solutions were prepared by appropriate dilution of the stock solutions with acetonitrile. Matrix-matched calibration curves were constructed by diluting the stock solutions with blank extract solutions obtained from pesticide-free fresh shiitake mushroom samples. All standard solutions were kept at 4 °C prior to analysis.

### Field trials

The field trials were conducted in accordance with the Guideline for the Testing of Pesticide Residue in Crops(NY/T 788-2018), issued by the Ministry of Agriculture and Rural Affairs of the People’s Republic of China^58^. The experimental substrate for mushroom cultivation was obtained from Shandong Xiaofeng Biotechnology Co., Ltd. This substrate consists of a mixture of wheat bran and sawdust, which has been inoculated with mycelium. Each mushroom cultivation bag measures 40 cm in length and 10 cm in diameter. These mushrooms were cultivated in a greenhouse in Jinan, Shandong Province, China, with fruiting initiated approximately one month after inoculation. The experiment was carried out in March 2025 under controlled environmental conditions, maintaining a temperature of approximately 20 °C and a relative humidity of around 80%. The experimental design included three replicate treatment plots receiving pesticide application and one untreated control plot. Each plot contained approximately 30 mushroom bags, and a buffer zone of at least 1 meter wide was established between plots to minimize cross-contamination. Both the pesticide dissipation dynamics study and the processing residue test were carried out in the same experimental area. According to the maximum recommended dosage on the product labels, four plant protection products were diluted in water at a spray volume of 600 L/ha and applied uniformly. Pesticide applications were performed once when shiitake mushrooms reached a height of 2 cm. No pesticides were applied in the control plot. For the dissipation study, approximately 500 g of shiitake mushrooms were randomly collected from each treated plot at 0 (2 h), 1, 3, 5, 7, and 10 days after application, with concurrent sampling from the control plot. Additionally, to ensure that the samples selected for processing trials contain quantifiable and representative pesticide residue levels, 6 kg of fresh shiitake mushrooms were separately collected from each treatment plot on day 3 post-application for the processing test. Dissipation and processing trials were conducted simultaneously in the same experimental plot to ensure consistent environmental conditions and enable direct comparison of residue behavior under identical field settings. All freshly collected samples were processed immediately on the day of harvest. All samples were homogenized and stored (wet) at −20 °C until further analysis. The plant protection products used in the experiment were as follows: (i) 20% dinotefuran suspensionconcentrate, applied at a dosage of 600 mL/ha; (ii) 72% pyraclostrobin water dispersible granule, applied at a dosage of 600 g/ha; (iii) 25% thiamethoxam water dispersible granule, applied at a dosage of 300 g/ha; (iv) 10% imidacloprid wettable powder, applied at a dosage of 20 kg/ha.

The degradation behaviors of dinotefuran, imidacloprid, thiamethoxam, pyraclostrobin, and their metabolites in shiitake mushrooms were well described by the first-order kinetic model and calculated using the following equations: *C*_t_ = *C*_0_ × *e*^-kt^ and *t*_1/2_ = (ln2)/*k*, where *C*_t_ represents the residue concentration at sampling time *t* (days), *C*_0_ denotes the initial residue concentration (0 day), *k* is the dissipation rate constant, and *t*_1/2_ refers to the pesticide half-life (days)^52,59^.

### Processing and sample preparation

In this study, the processing procedures were designed to simulate industrial and household practices, thereby closely replicating real-world conditions in a laboratory setting.

#### Washing

Approximately 0.5 kg of fresh shiitake mushrooms were soaked in 2 L tap water and manually cleaned; samples were collected after 5 min.

#### Boiling

Approximately 0.5 kg of fresh shiitake mushrooms were cut into 0.5 cm cubes and put into a pot containing 2 L of tap water. Subsequently, the water with the mushroom cubes was boiled for 10 min, after which samples were collected.

#### Pickling

Approximately 1.0 kg of fresh shiitake mushrooms were cut into 0.5 cm cubes, put into a glass bottle, mixed with 20 g of table salt, thoroughly stirred, and allowed to pickle for 12 h before sample collection.

#### Stir-frying

Approximately 1.0 kg of fresh shiitake mushrooms were cut into 0.5 cm cubes and placed into an electric frying pan (1000 W) preheated with 50 mL of peanut oil at 170 °C. Then, Stir-fry constantly for 15 min; samples were collected immediately after cooking.

#### Hot air drying

Approximately 1.0 kg of fresh shiitake mushrooms were placed in a forced-air circulation oven (Shanghai HASUC Instrument Manufacturing Co., Ltd., China) set at 50 °C with an airflow rate of 0.45 m/s and dried until the moisture content dropped below 10%, after which dried samples were collected^60^.

#### Sauce processing

Take approximately 1 kg of fresh shiitake mushrooms and remove any impurities or dirt^61^. Immerse the mushrooms in a color-protecting solution (0.2% ascorbic acid, 0.1% citric acid, 0.75% sodium chloride) for 10 min to prevent discoloration, followed by blanching in boiling tap water at 100 °C for 5 min. Subsequently, cut the mushrooms into small pieces and dry them in a hot air oven(Shanghai HASUCInstrument ManufacturingCo., Ltd., China) at 45 °C for 40 min. After drying, add 2.0 g spices and seasonings to the mushroom pieces and allow them to marinate at 25 °C for 30 min. Stir-fry the seasoned pieces in an electric frying pan (1000 W) at 170 °C for 5 min. Transfer the resulting mushroom sauce into glass bottles and process in a constant-temperature water bath at 100 °C for 20 min to expel residual air and achieve thermal sterilization. The final mushroom sauce product is thus obtained, and samples are collected for analysis.

#### Crisp processing

Cut approximately 1 kg of fresh shiitake mushrooms into slices 0.5 cm in thickness and blanch them in water at (96 ± 2)°C for 5 min^62^. Subsequently, immerse the mushrooms in cold water at 10 °C for 5 min. After draining, place the samples in a freezer at −10 °C for pre-freezing over a period of 12 h, followed by thawing at room temperature. Next, submerge all samples in sugar syrup—prepared in advance by mixing malt syrup and maltodextrin at a volume ratio of 5:2—for pickling over 24 h, after which remove the mushrooms. Following pickling, transfer the mushrooms to a vacuum freeze dryer (Nanjing Xiaoma Machinery Instrument Co., Ltd., China) and conduct freeze-drying at −18 °C under a vacuum pressure of 0.37 mbar for 48 h. Upon completion of drying, collect the resulting mushroom crisp samples.

All collected samples were homogenized and stored at −20 °C for subsequent analysis. The PF value was used to assess changes in pesticide residues during the processing of shiitake mushrooms. The PF is calculated using the following equation. PF = *C*_pp_/*C*_rac_, where *C*_pp_ is the residue concentration in processed products (PP) and *C*_rac_ is the residue concentration in the raw agricultural commodity (RAC). A PF greater than 1 indicates an increase in pesticide residues during processing, whereas a PF less than 1 indicates a reduction in pesticide residues.

To evaluate statistically significant variations in pesticide residue concentrations across different processing methods, data analysis was conducted using IBM SPSS version 19.0. Each experimental condition was replicated three times to ensure reliability. Differences in PF values were assessed via paired-sample *t*-tests, with statistical significance set at *P* < 0.05^32^.

### Extraction of pesticide residues

All collected matrices were analyzed in triplicate. Homogenized shiitake mushroom samples (5 g of fresh shiitake mushroom, 2 g of dried shiitake mushroom) were accurately weighed and placed in 50 mL centrifuge tubes. For the dried shiitake mushroom samples, an additional 5 mL of water should be added, followed by vortexing for 1 min. Then, 10 mL of acetonitrile should be added, and the mixture should be vigorously shaken for 5 min. Subsequently, the buffer-salt mixture was added to each tube, mixed vigorously for 1 min, and centrifuged at 4200 r/min for 5 min. Then, 6 mL of the supernatant was transferred to a cleanup tube containing PSA and MgSO_4_, shaken for 1 min, and centrifuged again under the same conditions. Finally, 1.5 mL of the resulting supernatant was filtered through a 0.22 μm nylon membrane and transferred into a vial for instrumental analysis.

### Apparatus and chromatographic conditions

The Agilent 1290 Infinity II ultra-high-performance liquid chromatograph (UPLC) coupled with an Agilent 6475 triple quadrupole mass spectrometer (MS/MS) (Agilent, CA, USA) was used for qualitative and quantitative analyses of dinotefuran, thiamethoxam, pyraclostrobin, and their metabolites. Chromatographic separation was achieved using a Poroshell 120 EC-C18 column (100 × 4.6 mm, 2.7 μm, Agilent) maintained at 40 °C, with a flow rate of 0.3 mL/min and an injection volume of 2 μL. The mobile phase consisted of water (Phase A) and acetonitrile (Phase B). Gradient elution was performed as follows: 0–0.2 min, 90% A; 0.2–4 min, linear gradient from 90% to 10% A; 4–7 min, 10% A; 7–10 min, linear gradient from 10% to 90% A, followed by equilibration. The ion source parameters were set as follows: gas temperature, 280 °C; drying gas flow rate, 11 L/min; nebulizer pressure, 35 psi; sheath gas temperature, 250 °C; sheath gas flow rate, 11 L/min; capillary voltage, 3500 V; and nozzle voltage, 1500 V. Detailed information regarding qualitative and quantitative ions, collision energy, fragmentor voltage, and ion polarity is provided in Table S5.

### Method validation

According to the EU guidance document SANTE/11312/2021 v2^37^, this analytical method was comprehensively validated with respect to linearity, matrix effect (ME), limit of quantification (LOQ), accuracy, and precision. All blank samples were analyzed to confirm the absence of target pesticide residues. Linearity was evaluated across a concentration range of 1–500 μg/L using six calibration levels (1.0, 5.0, 10.0, 50.0, 100.0, and 500.0 μg/L), based on both solvent-based and matrix-matched calibration curves. The LOQ was defined as the lowest spiked concentration that could be reliably detected and quantified in shiitake mushroom samples. The ME, which reflects signal suppression or enhancement due to co-extracted matrix components, was calculated according to the formula: ME (%) = [slope (matrix)/slope (solvent) − 1] × 100%^63^. Accuracy and precision were assessed through recovery experiments performed at three spiking levels (2 or 10, 100, and 1000 μg/kg), with five replicates per level.

### Risk assessment

When calculating pesticide residue levels, each pesticide is defined as the sum of its parent compound and its metabolites, expressed in terms of the parent pesticide. Therefore, the residue levels of dinotefuran, imidacloprid, and pyraclostrobin are calculated as follows. However, although clothianidin is a metabolite of thiamethoxam, according to the definition of the Joint Meeting on Pesticide Residues (JMPR), the residue levels of these two pesticides need to be calculated separately, and their residue levels are equivalent to the directly detected values.

1 $${{\rm{Dinotefuran}}}_{{\rm{sum}}}{={\rm{C}}}_{{\rm{dinotefuran}}}+(1{.284\times {\rm{C}}}_{{\rm{DN}}})+(1{.277\times {\rm{C}}}_{{\rm{UF}}})$$

2 $${{\rm{Imidacloprid}}}_{{\rm{sum}}}{={\rm{C}}}_{{\rm{imidacloprid}}}+(1{.642\times {\rm{C}}}_{6-{\rm{chloronicotinicacid}}})$$

3 $${{\rm{Pyraclostrobin}}}_{{\rm{sum}}}{={\rm{C}}}_{{\rm{pyraclostrobin}}}+(1{.084\times {\rm{C}}}_{{\rm{BF}}-500-3})$$

where *C*_dinotefuran_, *C*_DN_, *C*_UF_, *C*_imidacloprid_, *C*_6-chloronicotinicacid_, *C*_pyraclostrobin,_ and *C*_BF-500-3_ are the residue levels of dinotefuran, DN, UF, imidacloprid, 6-chloronicotinic acid, pyraclostrobin, and BF-500-3in shiitake mushrooms, respectively. According to the principle of maximum risk, if the pesticide residue content in the sample is below the LOQ, the LOQ value should be used in the calculation instead of the actual residue amount.

Since shiitake mushrooms are not suitable for raw consumption, they require thermal processing before consumption. Furthermore, the edible mushroom consumption data reported by the FAO/WHO of the United Nations pertain specifically to dried products rather than fresh ones. Therefore, this study assessed the chronic and acute dietary risks associated with consuming dried shiitake mushrooms in both children and the general population, following the FAO/WHO recommended methodology^56^. The national estimated daily intake (NEDI, μg/kg bw/day) and chronic risk quotient (RQc) were calculated using Eqs. (4) and (5), respectively, to evaluate the long-term dietary exposure risk to pesticide residues from dried shiitake mushroom consumption. Similarly, the international estimated short-term intake (IESTI, μg/kg bw/day) and acute risk quotient (RQa) were computed using Eqs. (6) and (7) to assess the potential acute dietary risk.

4 $${\rm{NEDI}}=({\rm{STMR}}\times {\rm{PF}}\times {\rm{F}})/{\rm{bw}}/1000$$

5 $${\rm{RQc}}={\rm{NEDI}}/{\rm{ADI}}$$

6 $${\rm{IESTI}}=({\rm{LP}}\times {\rm{HR}}\times {\rm{PF}})/{\rm{bw}}/1000$$

7 $${\rm{RQa}}={\rm{IESTI}}/{\rm{ARfD}}$$

where, STMR and HR is the supervised trials median and highest residue in shiitake mushrooms (μg/kg), respectively. PF is the processing factor for each pesticide. *F* is the average consumption of dried shiitake mushrooms (30.0 g/day)^64^; bw is the body weight of consumers (19 kg for 2–6-year-old children and 53.2 kg for the general population in China)^56^. LP is the large portion of dried shiitake mushrooms (12.71 g/day for children and 46.28 g/day for the general population)^56^. ADI is the acceptable daily intake of a given pesticide (μg/kg bw/day). ARfD is the acute reference dose of a given pesticide (μg/kg bw/day). RQ is the risk quotient; the larger the RQ values, the higher the health risk; if RQ > 1, it poses an unacceptable risk. The ADI and ARfD values for the pesticides were obtained from the JMPR report^38^. The ADI values for clothianidin, dinotefuran, imidacloprid, pyraclostrobin, and thiamethoxam are 100, 200, 60, 30, and 80 μg/kg bw, respectively. The ARfD values for clothianidin, dinotefuran, imidacloprid, pyraclostrobin, and thiamethoxam are 600, 1000, 400, 50, and 1000 μg/kg bw, respectively.

## Supplementary information

Supplementary information

## Data Availability

The datasets generated and/or analyzed during the current study are not publicly available due to commercial confidentiality and intellectual property considerations, but are available from the corresponding author on reasonable request.
