# Supplementary material for: Dissipation behavior and risk assessment of four pesticides in shiitake mushrooms from cultivation to processing
Source: NPJ Sci Food. 2026 Jul 6;10:333. doi: 10.1038/s41538-026-00975-5 (PMC13720072; doi:10.1038/s41538-026-00975-5)
Supplement: Supplementary file 1 [file 41538_2026_975_MOESM1_ESM.docx]

**Title: Dissipation and migration of four pesticides and their metabolites in shiitake mushroom from cultivation to postharvest processing and dietary risk assessment**

**npj Science of Food**

Jingyun Liang^1^, Shuai Guan^1^, Mengmeng Yan^1^, Junhua Liu^1^, Liping Fang^1^, Jiangsheng Mao^1✉^ , Zhan Dong^1✉^, Kai Cui^1^, Changhan Zhang^2^

^1^Institute of Agricultural Products Quality Safety and Standards, Shandong Academy of Agricultural Sciences, Jinan, Shandong 250100, People’s Republic of China

^2^College of Food Science and Bioengineering, Tianjin Agricultural University, Tianjin 300392, People’s Republic of China

**Corresponding authors** **at:** Institute of Agricultural Products Quality Safety and Standards, Shandong Academy of Agricultural Sciences, Jinan, China.

✉e-mail: maojiangsheng@163.com (J. Mao), zhandongsaas@163.com (Z. Dong).

**Supplementary**

**Table S1.** Linearity, matrix effects (MEs), average recoveries, and relative standard deviations (RSDs) for four pesticides and five metabolites in different samples.

| Compounds | Matrix | Regression equation | R^2^ | ME (%) | Average recovery (n=5), % (RSD, %) | | |
| --- | --- | --- | --- | --- | --- | --- | --- |
|  |  |  |  |  | 2 or 10 μg/kg | 100 μg/kg | 1000 μg/kg |
| Dinotefuran | acetonitrile | y = 85.3 x + 254 | 0.9990 | - | - | - | - |
|  | fresh | y = 89.8x + 959 | 0.9983 | 5.3 | 76±6.7 | 76±5.4 | 77±6.2 |
|  | dried | y = 94.4x + 672 | 0.9986 | 10.7 | 94±6.8 | 94±6.4 | 84±5.0 |
|  | sauce | y = 86.6x + 2,074 | 0.9986 | 1.5 | 102±10.7 | 105±6.7 | 79±6.6 |
| DN | acetonitrile | y = 6,310.2x + 4,053 | 0.9995 | - | - | - | - |
|  | fresh | y = 5,720.9x – 12,856 | 0.9981 | -9.3 | 76±6.0 | 77±3.4 | 78±5.1 |
|  | dried | y = 6,858.0 x + 4,650 | 0.9998 | 8.7 | 87±6.6 | 85±7.8 | 88±11.3 |
|  | sauce | y = 4,221.6x + 4,838 | 0.9995 | -33.1 | 78±3.1 | 80±4.5 | 80±2.0 |
| UF | acetonitrile | y = 1,326.2 x - 2,716 | 0.9990 | - | - | - | - |
|  | fresh | y = 1,121.5x - 791 | 0.9999 | -15.4 | 74±4.5 | 74±4.2 | 76±5.8 |
|  | dried | y = 356.3x – 1,986 | 0.9995 | -73.1 | 106±6.2 | 82±8.8 | 80±5.2 |
|  | sauce | y = 1,136.8x – 2,565 | 0.9993 | -14.3 | 103±5.5 | 79±3.4 | 91±5.2 |
| Imidacloprid | acetonitrile | y = 159.5x - 489 | 0.9969 | - | - | - | - |
|  | fresh | y = 97.0x + 385 | 0.9993 | -39.2 | 81±6.7 | 88±8.3 | 94±5.2 |
|  | dried | y = 138.4x - 117 | 0.9984 | -13.2 | 78±5.4 | 91±5.6 | 97±7.0 |
|  | sauce | y = 79.3x + 236 | 0.9994 | -50.3 | 111±6.5 | 101±7.1 | 94±5.4 |
| 6-chloronicotinic acid | acetonitrile | y = 9.1x - 11 | 0.9992 | - | - | - | - |
|  | fresh | y = 3.4x + 4 | 0.9974 | -62.6 | 75±4.3 | 78±4.2 | 82±7.1 |
|  | dried | y = 6.3x - 1 | 0.9987 | -30.8 | 94±9.6 | 76±4.0 | 81±2.6 |
|  | sauce | y = 2.4x + 2 | 0.9988 | -73.6 | 77±2.6 | 80±5.8 | 77±3.4 |
| Pyraclostrobin | acetonitrile | y = 2,640.0x + 6,799 | 0.9972 | - | - | - | - |
|  | fresh | y = 4,381.5x + 15,132 | 0.9960 | 66.0 | 98±10.1 | 102±6.9 | 87±8.1 |
|  | dried | y = 4,045.3x + 1,017 | 0.9999 | 53.2 | 84±7.5 | 92±7.5 | 90±7.0 |
|  | sauce | y = 1,631.3x + 442 | 0.9994 | -38.2 | 98±7.5 | 91±8.6 | 81±8.2 |
| BF-500-3 | acetonitrile | y = 2,432.0x + 6,555 | 0.9960 | - | - | - | - |
|  | fresh | y = 4,509.2x + 14,677 | 0.9961 | 85.4 | 97±12.7 | 106±8.1 | 85±5.5 |
|  | dried | y = 4,336.8x – 4,447 | 0.9987 | 78.3 | 90±8.5 | 90±8.0 | 87±6.3 |
|  | sauce | y = 1,730.1x – 1,798 | 0.9999 | -28.9 | 91±6.5 | 87±11.5 | 78±7.0 |
| Thiamethoxam | acetonitrile | y = 98.1x - 183 | 0.9992 | - | - | - | - |
|  | fresh | y = 64.5x + 18 | 0.9988 | -34.3 | 81±6.8 | 100±13.4 | 81±4.7 |
|  | dried | y = 80.9x - 21 | 0.9987 | -17.5 | 79±3.0 | 94±6.0 | 95±7.1 |
|  | sauce | y = 47.4x + 99 | 0.9996 | -51.7 | 88±6.3 | 93±4.5 | 86±6.7 |
| Clothianidin | acetonitrile | y = 174.0 x - 365 | 0.9990 | - | - | - | - |
|  | fresh | y = 132.7x - 594 | 0.9928 | -23.7 | 81±6.8 | 95±13.7 | 92±15.0 |
|  | dried | y = 146.0x + 5 | 0.9996 | -16.1 | 87±8.0 | 88±8.5 | 96±5.2 |
|  | sauce | y = 85.5x + 268 | 0.9987 | -50.9 | 78±6.8 | 92±8.5 | 90±6.4 |

**Table S2.** Physicochemical parameters of different pesticides used in the study. (molecular weight, MW; solubility in water, Sw, 20°C, mg/ L; melting point, Mp, °C; degradation point, Dp, °C; octanol–water partition coefficient, log *Kow*, pH = 7, 20°C; vapor pressure, 20°C, mPa).

| Pesticides | CAS | MW | Sw | Mp | Dp | log Kow | Vp |
| --- | --- | --- | --- | --- | --- | --- | --- |
| Dinotefuran | 165252-70-0 | 202.2 | 39800 | 107.5 | - | -0.55 | <1.7E-03 |
| Imidacloprid | 138261-41-3 | 255.7 | 610 | 144.0 | 230 | 0.57 | 4E-07 |
| Pyraclostrobin | 175013-18-0 | 387.8 | 1.9 | 63.7 | 200 | 3.99 | 2.6E-05 |
| Thiamethoxam | 153719-23-4 | 291.7 | 4100 | 139.1 | 147 | -0.13 | 6.6E-06 |
| Clothianidin | 210880-92-5 | 249.7 | 340 | 176.8 | - | 0.9 | 3.8E-08 |

**Table S3.** Chronic and acute risk quotients (RQs) of different pesticides for children and the general populations from dried shiitake mushrooms consumption.

| Pesticides | PHIs | Residue (μg/ kg) | NEDI (μg/ kg bw/d) | | RQc | | IESTI (μg/kg bw/d) | | RQa | |
| --- | --- | --- | --- | --- | --- | --- | --- | --- | --- | --- |
|  |  |  | 2-6 years  child | general population | 2-6 years  child | general population | 2-6 years  child | general population | 2-6 years  child | general population |
| Dinotefuran | 2h | 2310.57 | 15.688 | 5.603 | 0.078 | 0.028 | 6.646 | 8.643 | 0.007 | 0.009 |
|  | 1d | 1299.07 | 8.820 | 3.150 | 0.044 | 0.016 | 3.737 | 4.859 | 0.004 | 0.005 |
|  | 3d | 443.61 | 3.012 | 1.076 | 0.015 | 0.005 | 1.276 | 1.659 | 0.001 | 0.002 |
|  | 5d | 197.10 | 1.338 | 0.478 | 0.007 | 0.002 | 0.567 | 0.737 | 0.001 | 0.001 |
|  | 7d | 53.48 | 0.363 | 0.130 | 0.002 | 0.001 | 0.154 | 0.200 | < 0.001 | < 0.001 |
|  | 10d | 14.12 | 0.096 | 0.034 | < 0.001 | < 0.001 | 0.041 | 0.053 | < 0.001 | < 0.001 |
| Imidacloprid | 2h | 12203.21 | 5.780 | 2.064 | 0.096 | 0.034 | 2.449 | 3.185 | 0.006 | 0.008 |
|  | 1d | 6399.61 | 3.031 | 1.083 | 0.051 | 0.018 | 1.284 | 1.670 | 0.003 | 0.004 |
|  | 3d | 4167.65 | 1.974 | 0.705 | 0.033 | 0.012 | 0.836 | 1.088 | 0.002 | 0.003 |
|  | 5d | 859.57 | 0.407 | 0.145 | 0.007 | 0.002 | 0.173 | 0.224 | < 0.001 | 0.001 |
|  | 7d | 333.64 | 0.158 | 0.056 | 0.003 | 0.001 | 0.067 | 0.087 | < 0.001 | < 0.001 |
|  | 10d | 44.89 | 0.021 | 0.008 | < 0.001 | < 0.001 | 0.009 | 0.012 | < 0.001 | < 0.001 |
| Pyraclostrobin | 2h | 4189.50 | 0.662 | 0.236 | 0.022 | 0.008 | 0.280 | 0.364 | 0.006 | 0.007 |
|  | 1d | 2777.94 | 0.439 | 0.157 | 0.015 | 0.005 | 0.186 | 0.242 | 0.004 | 0.005 |
|  | 3d | 1670.28 | 0.264 | 0.094 | 0.009 | 0.003 | 0.112 | 0.145 | 0.002 | 0.003 |
|  | 5d | 456.59 | 0.072 | 0.026 | 0.002 | 0.001 | 0.031 | 0.040 | 0.001 | 0.001 |
|  | 7d | 274.41 | 0.043 | 0.015 | 0.001 | 0.001 | 0.018 | 0.024 | < 0.001 | < 0.001 |
|  | 10d | 143.35 | 0.023 | 0.008 | 0.001 | < 0.001 | 0.010 | 0.012 | < 0.001 | < 0.001 |
| Thiamethoxam | 2h | 2884.83 | 1.822 | 0.651 | 0.023 | 0.008 | 0.772 | 1.004 | 0.001 | 0.001 |
|  | 1d | 1989.03 | 1.256 | 0.449 | 0.016 | 0.006 | 0.532 | 0.692 | 0.001 | 0.001 |
|  | 3d | 1027.38 | 0.649 | 0.232 | 0.008 | 0.003 | 0.275 | 0.357 | < 0.001 | < 0.001 |
|  | 5d | 477.96 | 0.302 | 0.108 | 0.004 | 0.001 | 0.128 | 0.166 | < 0.001 | < 0.001 |
|  | 7d | 222.72 | 0.141 | 0.050 | 0.002 | 0.001 | 0.060 | 0.078 | < 0.001 | < 0.001 |
|  | 10d | 61.34 | 0.039 | 0.014 | < 0.001 | < 0.001 | 0.016 | 0.021 | < 0.001 | < 0.001 |
| Clothianidin | 2h | 17.00 | 0.008 | 0.003 | < 0.001 | < 0.001 | 0.003 | 0.004 | < 0.001 | < 0.001 |
|  | 1d | 41.82 | 0.020 | 0.007 | < 0.001 | < 0.001 | 0.008 | 0.011 | < 0.001 | < 0.001 |
|  | 3d | 128.24 | 0.061 | 0.022 | < 0.001 | < 0.001 | 0.026 | 0.033 | < 0.001 | < 0.001 |
|  | 5d | 28.22 | 0.013 | 0.005 | < 0.001 | < 0.001 | 0.006 | 0.007 | < 0.001 | < 0.001 |
|  | 7d | 14.03 | 0.007 | 0.002 | < 0.001 | < 0.001 | 0.003 | 0.004 | < 0.001 | < 0.001 |
|  | 10d | 5.42 | 0.003 | 0.001 | < 0.001 | < 0.001 | 0.001 | 0.001 | < 0.001 | < 0.001 |

**Table S4.** Chronic and acute risk quotients (RQs) of different pesticides for children and the general populations from shiitake crisps consumption.

| Pesticides | PHIs | | Residue (μg/ kg) | NEDI (μg/ kg bw/d) | | RQc | | IESTI (μg/kg bw/d) | | RQa | |
| --- | --- | --- | --- | --- | --- | --- | --- | --- | --- | --- | --- |
|  |  |  |  | 2-6 years  child | general population | 2-6 years  child | general population | 2-6 years  child | general population | 2-6 years  child | general population |
| Dinotefuran | 2h | 2310.57 | | 9.121 | 3.257 | 0.046 | 0.016 | 3.864 | 5.025 | 0.004 | 0.005 |
|  | 1d | 1299.07 | | 5.128 | 1.831 | 0.026 | 0.009 | 2.173 | 2.825 | 0.002 | 0.003 |
|  | 3d | 443.61 | | 1.751 | 0.625 | 0.009 | 0.003 | 0.742 | 0.965 | 0.001 | 0.001 |
|  | 5d | 197.10 | | 0.778 | 0.278 | 0.004 | 0.001 | 0.330 | 0.429 | < 0.001 | < 0.001 |
|  | 7d | 53.48 | | 0.211 | 0.075 | 0.001 | < 0.001 | 0.089 | 0.116 | < 0.001 | < 0.001 |
|  | 10d | 14.12 | | 0.056 | 0.020 | < 0.001 | < 0.001 | 0.024 | 0.031 | < 0.001 | < 0.001 |
| Imidacloprid | 2h | 12203.21 | | 48.171 | 17.204 | 0.803 | 0.287 | 20.408 | 26.540 | 0.051 | 0.066 |
|  | 1d | 6399.61 | | 25.262 | 9.022 | 0.421 | 0.150 | 10.702 | 13.918 | 0.027 | 0.035 |
|  | 3d | 4167.65 | | 16.451 | 5.875 | 0.274 | 0.098 | 6.970 | 9.064 | 0.017 | 0.023 |
|  | 5d | 859.57 | | 3.393 | 1.212 | 0.057 | 0.020 | 1.438 | 1.869 | 0.004 | 0.005 |
|  | 7d | 333.64 | | 1.317 | 0.470 | 0.022 | 0.008 | 0.558 | 0.726 | 0.001 | 0.002 |
|  | 10d | 44.89 | | 0.177 | 0.063 | 0.003 | 0.001 | 0.075 | 0.098 | < 0.001 | < 0.001 |
| Pyraclostrobin | 2h | 4189.50 | | 15.876 | 5.670 | 0.529 | 0.189 | 6.726 | 8.747 | 0.135 | 0.175 |
|  | 1d | 2777.94 | | 10.527 | 3.760 | 0.351 | 0.125 | 4.460 | 5.800 | 0.089 | 0.116 |
|  | 3d | 1670.28 | | 6.329 | 2.261 | 0.211 | 0.075 | 2.682 | 3.487 | 0.054 | 0.070 |
|  | 5d | 456.59 | | 1.730 | 0.618 | 0.058 | 0.021 | 0.733 | 0.953 | 0.015 | 0.019 |
|  | 7d | 274.41 | | 1.040 | 0.371 | 0.035 | 0.012 | 0.441 | 0.573 | 0.009 | 0.011 |
|  | 10d | 143.35 | | 0.543 | 0.194 | 0.018 | 0.006 | 0.230 | 0.299 | 0.005 | 0.006 |
| Thiamethoxam | 2h | 2884.83 | | 18.220 | 6.507 | 0.228 | 0.081 | 7.719 | 10.038 | 0.008 | 0.010 |
|  | 1d | 1989.03 | | 12.562 | 4.487 | 0.157 | 0.056 | 5.322 | 6.921 | 0.005 | 0.007 |
|  | 3d | 1027.38 | | 6.489 | 2.317 | 0.081 | 0.029 | 2.749 | 3.575 | 0.003 | 0.004 |
|  | 5d | 477.96 | | 3.019 | 1.078 | 0.038 | 0.013 | 1.279 | 1.663 | 0.001 | 0.002 |
|  | 7d | 222.72 | | 1.407 | 0.502 | 0.018 | 0.006 | 0.596 | 0.775 | 0.001 | 0.001 |
|  | 10d | 61.34 | | 0.387 | 0.138 | 0.005 | 0.002 | 0.164 | 0.213 | < 0.001 | < 0.001 |
| Clothianidin | 2h | 17.00 | | 0.086 | 0.031 | 0.001 | < 0.001 | 0.036 | 0.047 | < 0.001 | < 0.001 |
|  | 1d | 41.82 | | 0.211 | 0.075 | 0.002 | 0.001 | 0.090 | 0.116 | < 0.001 | < 0.001 |
|  | 3d | 128.24 | | 0.648 | 0.231 | 0.006 | 0.002 | 0.275 | 0.357 | < 0.001 | 0.001 |
|  | 5d | 28.22 | | 0.143 | 0.051 | 0.001 | 0.001 | 0.060 | 0.079 | < 0.001 | < 0.001 |
|  | 7d | 14.03 | | 0.071 | 0.025 | 0.001 | < 0.001 | 0.030 | 0.039 | < 0.001 | < 0.001 |
|  | 10d | 5.42 | | 0.027 | 0.010 | < 0.001 | < 0.001 | 0.012 | 0.015 | < 0.001 | < 0.001 |

**Table S5.** The MRM parameters of four pesticides and five metabolites for UPLC-MS/MS detection.

| Compounds | Chemical formula | Retention time (min) | Quantitative ion (m/z) | Qualitative ion (m/z) | CE (V)  Quan/qual | Fragmentor (V) | Ion |
| --- | --- | --- | --- | --- | --- | --- | --- |
| Dinotefuran | C_7_H_14_N_4_O_3_ | 1.863 | 203.1 / 129.2 | 203.1 / 87.1 | 20 / 12 | 135 | positive |
| DN | C_7_H_15_N_3_O | 0.931 | 158.2 / 102.1 | 158.2 / 57.1 | 35 / 15 | 135 | positive |
| UF | C_7_H_14_N_2_O_2‌_ | 0.939 | 159.1 / 102.0 | 159.1 / 67.0 | 17 / 8 | 135 | positive |
| Imidacloprid | C_9_HClN_5_O_2_ | 2.728 | 256.1 / 209.0 | 256.1 / 175.2 | 21 / 16 | 150 | positive |
| 6-chloronicotinic acid | C_6_H_4_ClNO_2_ | 2.583 | 156.0 / 122.0 | 156.0 / 111.9 | 8 / 21 | 110 | negative |
| Pyraclostrobin | C_19_H_18_ClN_3_O_4_ | 4.743 | 388.1 / 194.0 | 388.1 / 163.0 | 25 / 8 | 110 | positive |
| BF-500-3 | C_18_H_16_ClN_3_O_3_ | 4.727 | 358.1 / 164.0 | 358.1 / 132.0 | 40 / 10 | 110 | positive |
| Thiamethoxam | C_8_H_10_ClN_5_O_3_S‌ | 2.403 | 292.0 / 211.0 | 292.0 / 181.0 | 20 / 5 | 135 | positive |
| Clothianidin | C_6_H_8_ClN_5_O_2_S | 2.629 | 250.1 / 169.2 | 250.1 / 132.1 | 21 / 12 | 135 | positive |
